# Atherosclerosis differentially affects calcium signalling in endothelial cells from aortic arch and thoracic aorta in Apolipoprotein E knockout mice

**DOI:** 10.14814/phy2.12171

**Published:** 2014-10-24

**Authors:** Clodagh Prendergast, John Quayle, Theodor Burdyga, Susan Wray

**Affiliations:** 1 Department of Cellular & Molecular Physiology, Institute of Translational Medicine, University of Liverpool, Liverpool, UK

**Keywords:** Apolipoprotein‐E knockout mice, calcium signalling, endothelium, hypercholesterolemia

## Abstract

Apolipoprotein‐E knockout (ApoE^−/−^) mice develop hypercholesterolemia and are a useful model of atherosclerosis. Hypercholesterolemia alters intracellular Ca^2+^ signalling in vascular endothelial cells but our understanding of these changes, especially in the early stages of the disease process, is limited. We therefore determined whether carbachol‐mediated endothelial Ca^2+^ signals differ in plaque‐prone aortic arch compared to plaque‐resistant thoracic aorta, of wild‐type and ApoE^−/−^ mice, and how this is affected by age and the presence of hypercholesterolemia. The extent of plaque development was determined using *en‐face* staining with Sudan IV. Tissues were obtained from wild‐type and ApoE^−/−^ mice at 10 weeks (pre‐plaques) and 24 weeks (established plaques). We found that even before development of plaques, significantly increased Ca^2+^ responses were observed in arch endothelial cells. Even with aging and plaque formation, ApoE^−/−^ thoracic responses were little changed, however a significantly enhanced Ca^2+^ response was observed in arch, both adjacent to and away from lesions. In wild‐type mice of any age, 1–2% of cells had oscillatory Ca^2+^ responses. In young ApoE^−/−^ and plaque‐free regions of older ApoE^−/−^, this is unchanged. However a significant increase in oscillations (~13–15%) occurred in thoracic and arch cells adjacent to lesions in older mice. Our data suggest that Ca^2+^ signals in endothelial cells show specific changes both before and with plaque formation, that these changes are greatest in plaque‐prone aortic arch cells, and that these changes will contribute to the reported deterioration of endothelium in atherosclerosis.

## Abstract (precis)

We have investigated aortic endothelial cell calcium signalling changes in the Apolipoprotein E knockout mouse model of atherosclerosis. Our data show that calcium signals in endothelial cells undergo specific changes both before and with plaque formation, that these changes are greater in plaque‐prone aortic arch than in plaque‐resistant thoracic aorta, and that these changes will contribute to the reported deterioration of endothelium in atherosclerosis.

## Introduction

The vascular endothelium plays a vital role in the maintenance of vascular health and homeostasis. In addition to regulating vascular tone and blood flow through the production of nitric oxide and other endothelial‐derived relaxing factors (EDRF), endothelial cells regulate cellular adhesion, thromboresistance, smooth muscle cell proliferation and inflammation. Endothelial dysfunction therefore is implicated in many pathological conditions such as atherosclerosis, hypertension, sepsis and inflammatory conditions (Galley and Webster 2004; Page and Liles 2013).

Apolipoprotein‐E knockout (ApoE^−/−^) mice, a widely used model of atherosclerosis, spontaneously develop hypercholesterolemia and atherosclerotic plaques when fed a normal diet (Plump et al. 1992; Zhang et al. 1992; Nakashima et al. 1994). The cellular composition of the atherosclerotic plaques is remarkably similar to those of humans (Reddick et al. 1994).

Decreased vascular function occurs in atherosclerosis (Verbeuren et al. 1986; Forstermann et al. 1988; Ragazzi et al. 1989; Fontes Ribeiro et al. 1998). In studies of human vessels, decreased function is reported prior to plaque formation (Creager et al. 1990; Zeiher et al. 1991; Reddy et al. 1994).

In ApoE^−/−^ mice, an endothelial dysfunction, characterised by reduced ACh‐mediated relaxation, has been reported in plaque‐laden regions of the aorta, for example, in mice on a western‐style diet and in older ApoE^−/−^ mice on a normal diet (Deckert et al. 1999; Yang et al. 1999). Crauwels et al. (2003) showed that ACh‐mediated relaxation was unaltered in young ApoE^−/−^ mice prior to plaque development, although the basal availability of NO appeared to be compromised before the onset of disease. In old ApoE^−/−^ mice, endothelial dysfunction occurs but was strictly correlated with the development and size of plaques, with plaque‐free regions exhibiting normal behaviour (Bonthu et al. 1997; Deckert et al. 1999; Yaghoubi et al. 2000; Crauwels et al. 2003). Our previous studies in ApoE^−/−^ mice (Prendergast et al. 2014) used confocal microscopy and demonstrated a significantly increased intracellular Ca^2+^ response to a single maximal concentration of CCh in young ApoE^−/−^ aortic endothelial cells compared to wild‐type, but despite this, we observed an impaired relaxation to carbachol. These changes were apparent before plaque development, as well as when plaques were well established.

Calcium signalling is fundamental to vascular endothelial cell signalling and we need a better understanding of how it is affected by dyslipidemia. The Ca^2+^ response observed when endothelial cells are stimulated with agonist is complex and depends on both Ca^2+^ release and entry: IP_3_‐mediated release of Ca^2+^ from the intracellular store and entry of external Ca^2+^ via store‐operated Ca^2+^ channels, receptor‐operated channels or non‐selective cation channels (Wang et al. 1995; Wang and van Breemen 1997; Nilius and Droogmans 2001; Boittin et al. 2008). Interplay between ER Ca^2+^ release and uptake (via SERCA) and plasma membrane Ca^2+^ movements, shapes the Ca^2+^ signal amplitude and duration and also frequency, often referred to as Ca^2+^ oscillations (Nilius and Droogmans 2001). Muscarinic receptors (and many other GPCRs), G proteins, eNOS, plasma membrane Ca^2+^‐ATPase and Ca^2+^ entry channels, have all been shown to be localised to the plasma membrane microdomains known as caveolae (Nilius and Droogmans 2001). Caveolae are dynamic signalling domains and the function of their associated signalling molecules can be modulated by altered cholesterol levels, as a result of disease (Noble et al. 2006; Xu et al. 2008; Pavlides et al. 2012) or through experimental manipulation (Dreja et al. 2002; Frank et al. 2004; Smith et al. 2005; Prendergast et al. 2010). In ApoE^−/−^ mice, the 5‐fold increase in total plasma cholesterol levels (Zhang et al. 1992) has been shown to alter several aspects of the Ca^2+^ signalling pathways in endothelial and smooth muscle cells (Van Assche et al. 2007; Fransen et al. 2008; Ewart et al. 2014; Prendergast et al. 2014). Here, we examine the changes occurring in the Ca^2+^ signalling pathways in endothelial cells, as a result of chronically elevated cholesterol and development of atherosclerotic plaques, and how these affect the nature of the Ca^2+^ signals, particularly oscillations.

In this study we have used confocal microscopy to examine changes in intracellular Ca^2+^ in aortic endothelial cells from young and old WT and ApoE^−/−^ mice. We characterise the CCh dose‐response curve, in order to obtain both amplitude and EC_50_ data and correlate the responses with the degree of plaque progression in young 10 week ApoE^−/−^ mice (before overt plaques) and old 24 week ApoE^−/−^ mice (established plaques), and compare the data to that of WT controls.

Endothelial cells sense the shear stress generated by blood flow and their elicited signals evoke a cellular response (Ando and Yamamoto 2013). Impairment of this response has been associated with the development of vascular diseases including atherosclerosis. Furthermore it has been reported that early atherosclerotic lesions often develop in areas characterised by turbulent blood flow; that is curvatures, bifurcations and branch points. Atherosclerosis‐resistant regions are characterised by laminar blood flow (Ku et al. 1985; Berceli et al. 1990; Moore et al. 1994; Deng et al. 1995). We have therefore compared Ca^2+^ responses in the straight thoracic aorta with those in the more plaque‐prone aortic arch in WT mice, to investigate if flow differences affect Ca^2+^ signalling in endothelial cells, as this appears to have been little investigated previously. We have also compared data obtained in WT and the ApoE^−/−^ mice. Furthermore in older mice, where plaques were present, measurements were taken from endothelial cells both immediately adjacent to the plaques and at a distance, in order to determine whether the changes in Ca^2+^ response were dependent upon or altered by proximity to areas of plaque.

The aims of this study were therefore to: (1) determine whether the endothelial Ca^2+^ signals in response to CCh differ in aortic arch and thoracic aorta of WT mice and how this is affected by the presence of hypercholesterolemia, (2) determine the time course of changes in Ca^2+^ signals in WT and ApoE^−/−^ mice by studying different age groups and a range of agonist concentrations, (3) investigate if endothelial Ca^2+^ signals were affected by plaque formation, (4) test the hypothesis that Ca^2+^ signalling changes were greater in endothelial cells from aortic arch compared to thoracic aorta and (5) test the hypothesis that Ca^2+^ signals become more oscillatory with aging in ApoE^−/−^ but not WT mice.

## Materials and Methods

### Mice

Apolipoprotein E knockout mice (homozygotes) were obtained from Charles River and a breeding colony established in‐house. Wild‐type C57BL/6J mice were obtained from Charles River as required. All were maintained on a normal chow diet. Young (8–10 weeks) and old (22–24 weeks) male mice were used in experiments.

### Ethical approval

Mice were anaesthetized (CO_2_) and humanely killed by cervical dislocation in accordance with Schedule 1 of the UK Animals (Scientific Procedures) Act of 1986.

### Plaque assessment

The presence or absence of plaques in the aorta of WT and ApoE^−/−^ mice was confirmed using the lipophilic dye Sudan IV (Frank et al. 2004), which stains lipid deposits red. The aortic arch (from the top of the innominate artery branch down to 3 mm below the branching point of the subclavian artery) from 10 to 24 week old WT and ApoE^−/−^ mice was removed and fixed in 10% neutral buffered formalin for 24 h. After this, tissues were rinsed ×2 in PBS and stored in PBS at 4°C until the staining procedure was carried out as follows: Each aorta was washed in 70% EtOH for 5 min, transferred to the Sudan IV solution (composition (1L): 5 g Sudan IV, 500 mL acetone, 500 mL 70% EtOH) and gently agitated for 15 min and then destained in 80% EtOH for a further 5 min. Aortae were rinsed in PBS, dissected open along the outer edge of the arch and pinned out in a dissection dish containing PBS. Images were obtained using a dissecting microscope mounted with a Leica camera and plaque size assessed as a percentage of total surface area using ImageJ software. Plaques were not systematically investigated in the less plaque‐prone thoracic aorta, as they occur at too low a frequency.

### Confocal microscopy

The heart, with thoracic aorta attached, was removed and placed into a physiological salt solution of composition (mmol/L): NaCl 154, KCl 5.6, MgSO_4_7H_2_O 1.2, HEPES 10.9, Glucose 8, CaCl_2_ 2 (adjusted to pH 7.4). The aorta was cleared of adhering tissue, divided into arch and thoracic sections and incubated with 23 μmol/L Fluo‐4 AM for 2 h at room temperature in the presence of 0.25% of the non‐ionic detergent Pluronic F‐127 and subsequently cut into strips (~1 × 6 mm). The tissue was then placed in physiological salt solution to allow de‐esterification of the dye. Strips were mounted, endothelium face down, under a small amount of isometric tension between two fixed aluminum foil clips at the bottom of the chamber, on the stage of an Olympus inverted microscope. The chamber was perfused with physiological salt solution at a constant flow rate (1 mL/min) and maintained at 30°C. Experiments were performed using an Ultraview LCI spinning (Nipkow) disc, wide‐field confocal microscope (Perkin Elmer, Cambridge, UK), equipped with an Orca ER cooled CCD camera (Hamamatsu Photonics, Welwyn Garden City, UK) and a 20× objective (N.A. 0.7; see Burdyga et al. 2003). Mean fluorescence intensity was measured on‐line from regions of interest drawn over individual cells using UltraView software. Healthy‐looking, well‐loaded cells were chosen for analysis, with between 5 and 16 individual cells analysed per tissue strip. Movement artifacts were rarely a problem when measuring from individual cells in a vessel under isometric tension. However, if substantial movement occurred, measurements were not made. The numerical data obtained were saved to an ASCII file for further analysis using Origin 7.0 software. The amplitude of the [Ca^2+^]_i_ signal was expressed as a normalised pseudo ratio of Fluo‐4 fluorescence (F/F_0_). Once a stable baseline signal was established, the agonist carbachol was applied at 0.3, 1, 3 and 10 μmol/L to obtain a non‐cumulative dose‐response curve in both arch and thoracic aorta segments. The resulting Ca^2+^ signals were measured in terms of the amplitude of the initial peak response, the amplitude of the secondary plateau and the number of cells responding to CCh with an oscillatory Ca^2+^ response. Dose‐response curve data collected from plaque‐laden vessels was obtained from individual cells either immediately adjacent to plaques or in plaque‐free regions (no plaque present on entire strip), in order to compare whether this altered the Ca^2+^ signal observed. Similarly, Ca^2+^ oscillation data was collected from either totally plaque‐free strips or adjacent to plaques (either immediately adjacent or with plaque within the field of view). Data on Ca^2+^ oscillations from thoracic or arch regions were obtained and pooled with previously obtained data from WT (*n* = 11 young, *n* = 6 old) and ApoE^−/−^ (*n* = 9 young, *n* = 5 old) animals, which had not previously been divided into region or proximity to plaque (Prendergast et al. 2014).

### Drugs and solutions

Unless otherwise specified, chemicals were obtained from Sigma (UK). Pluronic‐F127 and Fluo‐4 AM were obtained from Invitrogen.

### Statistics

Data are presented as mean ± SEM, where *n* = number of cells and *N* = number of mice. Statistical analysis and curve fitting were carried out using Graphpad Prism 5. The Student's *t* test and one‐way ANOVA were used for statistical comparisons. A value of *P *<**0.05 was considered significant. In some cases, where Graphpad prism was unable to fit a curve to the mean data, a constraint was applied to the top of the curve (set at a value equal to 11% above the response obtained with 10 μmol/L CCh), which allowed curve‐fitting to proceed and an EC_50_ estimate to be obtained. This value was chosen by examining all the examples where curves were successfully fitted to the experimental data and comparing the size of the response measured with 10 μmol/L CCh with the calculated maximum of the fitted curve, i.e. on average, the response to 10 μmol/L CCh was determined to be just 11% below the maximum response.

## Results

**Figure 1.**
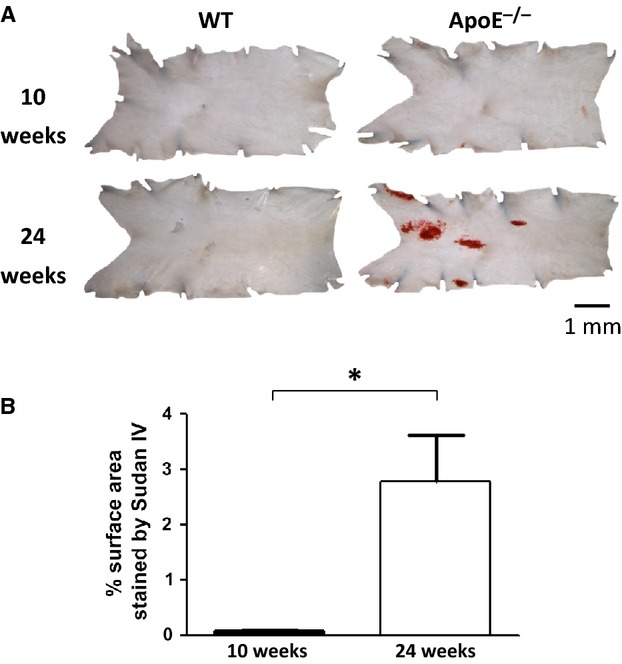
Sudan IV staining of atherosclerotic plaques. (A) Sections of WT and ApoE^−/−^ aorta from 10 to 24 week old animals. Plaque‐laden regions have been stained red with Sudan IV. (B) Percentage of total surface area covered in plaque. *P < 0.05

No WT mice, young or old, exhibited atherosclerotic plaques in the aortic arch. This was confirmed by staining with Sudan IV (Fig. 1A, 10 weeks, *N* = 10; 24 weeks, *N* = 5). In young ApoE^−/−^ mice, no plaques were visible by eye, and Sudan IV staining revealed either no plaque or barely visible tiny areas (*N* = 10). In older ApoE^−/−^ mice, all aortae exhibited clear atherosclerotic plaques (all visible by eye and confirmed using Sudan IV staining, *N* = 9, Fig. 1A), with significantly more plaque present at 24 weeks than 10 weeks (*P* = 0.003). The percentage of the total surface area covered by plaque was calculated to be: 10 weeks, 0.08 ± 0.02% and 24 weeks, 2.8 ± 0.8%; Fig. 1B). Plaques were not systematically investigated in the less plaque‐prone thoracic aorta, as they occur at too low a frequency (Nakashima et al. 1994; Van Assche et al. 2011).

### Overview of endothelial cell [Ca^2+^]_i_ response

**Figure 2.**
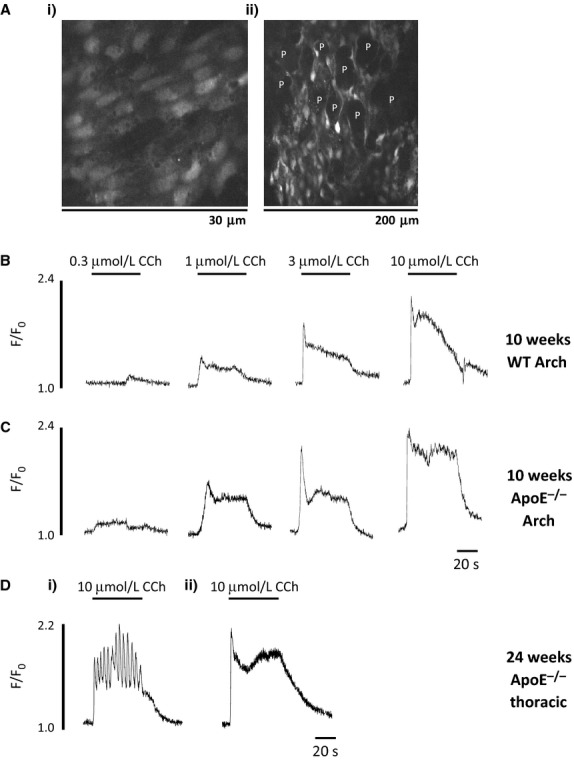
Calcium response to carbachol in aortic endothelial cells. (A) Confocal images of Fluo‐4 AM‐loaded ApoE^−/−^ endothelial cells from i) 24 week old plaque‐free aortic arch and ii) 24 week old plaque‐laden aortic arch, stimulated with 10 μmol/L CCh. Dark patches marked with P are plaque. Experimental traces showing CCh concentration‐response curves in endothelial cells from 10 week old (B) WT and (C) ApoE^−/−^ aortic arch sections. (D) Experimental traces showing (i) an oscillatory and (ii) a non‐oscillatory Ca^2+^ response to 10 μmol/L CCh in thoracic endothelial cells adjacent to plaque, from 24 week old ApoE^−/−^ mice.

Using confocal microscopy we observed that the endothelial layer was intact following tissue loading and preparation (Fig. 2Ai). Morphologically the ApoE^−/−^ endothelial cells appeared normal and were indistinguishable from WT cells. The effects of age, hypercholesterolemia and plaque formation on CCh‐mediated intracellular Ca^2+^ signals (0.3–10 μmol/L) were investigated in endothelial cells from arch and thoracic sections of the aorta. Figure 2Aii demonstrates that we can successfully visualise and measure changes in intracellular Ca^2+^ in endothelial cells that are immediately adjacent to areas of plaque (dark, spherical, unstained regions), as well as plaque‐free regions (Fig. 2Ai).

In all tissues CCh produced a concentration‐dependent rise in intracellular Ca^2+^ and typical responses from 10 week old mice are shown in Figure 2B and C. The response was characterised by a rapid upstroke of intracellular Ca^2+^ followed by a sustained plateau response in both WT and ApoE^−/−^ cells (Fig. 2B and C). Consistent with our previous data (Prendergast et al. 2014), we noted that ApoE^−/−^ preparations can respond to CCh by producing an oscillatory rather than sustained Ca^2+^ response (see Table 1 and Fig. 2D for a comparison of the oscillatory (1) and sustained (2) responses to CCh in old ApoE^−/−^ endothelial cells. These traces were obtained from cells adjacent to plaque, but the oscillatory responses, when they occur in plaque‐free areas, look similar). As noted earlier, the components and hence shape of the endothelial Ca^2+^ signal elicited by carbachol depends on several mechanisms, which may be affected differently by age and dyslipidemia. We therefore went on to compare the Ca^2+^ responses in terms of their initial peak response, the magnitude of the plateau phase and the percentage of cells that respond to CCh with an oscillatory Ca^2+^ response, in the different age groups, between WT and ApoE^−/−^ mice, and between endothelial cells in the arch and thoracic aorta.

**Table 1.** Percentage of endothelial cells responding to CCh with an oscillatory Ca^2+^ response.

	10 weeks					24 weeks				
	Tissue	Plaque status	% oscillations	*n* (cells)	*N*	Tissue	Plaque status	% oscillations	*n* (cells)	*N*
WT	Thoracic	Plaque‐free	1.5 ± 0.8	523	14	Thoracic	Plaque‐free	2.0 ± 0.7	219	4
	Arch	Plaque‐free	0.8 ± 0.7	446	8	Arch	Plaque‐free	0.5 ± 0.5	263	4
ApoE^−/−^	Thoracic	Plaque‐free	4.9 ± 2.5	564	13	Thoracic	Plaque‐free	4.5 ± 1.8	594	8
							Plaque	13.4 ± 3.7	685	8
	Arch	Plaque‐free	5.9 ± 2.3	646	12	Arch	Plaque‐free	1.5 ± 0.5	393	6
							Plaque	15.6 ± 5.5	649	11

### Regional dependency of CCh response: Thoracic versus arch

#### 10 week old mice

**Figure 3.**
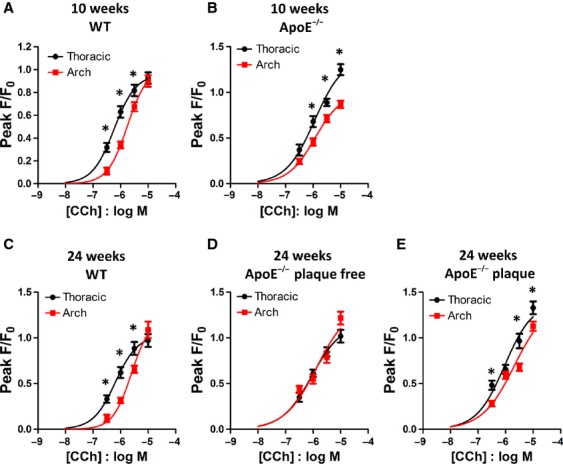
Regional dependency of calcium signalling in 10 and 24 week old WT and ApoE^−/−^ mice. Carbachol concentration‐response curves in endothelial cells from thoracic (●) and aortic arch () sections of (A) 10 week old WT mice, (B) 10 week old ApoE^−/−^ mice, (C) 24 week old WT mice, (D) 24 week old ApoE^−/−^ mice (plaque‐free) and (E) 24 week old ApoE^−/−^ mice (plaque‐laden). *P < 0.05

As shown in Figure 3, significant differences in Ca^2+^ signals were found between thoracic and arch regions of the aorta. In 10 week old WT mice, the amplitude of the Ca^2+^ response to CCh and the pEC_50_ was different in thoracic and arch sections of the aorta. In thoracic aorta, the amplitude of the response was greater (at 0.3, 1 & 3 μmol/L CCh) and the pEC_50_ more potent (peak data: pEC_50_ thoracic, 6.23 ± 0.10 vs. pEC_50_ arch, 5.75 ± 0.11, *N* = 4–5 mice, *P* = 0.014, Fig. 3A). In 10 week old ApoE^−/−^ sections, the amplitude of the peak Ca^2+^ response was again larger in thoracic aorta than aortic arch (at all concentrations of CCh), however there was no difference in pEC_50_ (peak data: pEC_50_ thoracic, 5.94 ± 0.19 vs. pEC_50_ arch, 5.93 ± 0.16, *N* = 5–7 mice, Fig. 3B).

### 24 week old mice

Similar to the 10 week old mice, the amplitude of the peak Ca^2+^ response was greater in WT thoracic aorta than WT arch (at 0.3, 1 & 3 μmol/L CCh) and pEC_50_ was more potent (peak data: pEC_50_ thoracic, 6.19 ± 0.13 versus pEC_50_ arch, 5.61 ± 0.15, *N* = 4 mice, *P* = 0.025, Fig. 3C). Plaques were established in ApoE^−/−^ mice of this age, so measurements were made in endothelial cells both adjacent to and away from areas of plaque. In 24 week ApoE^−/−^ mice and in cells adjacent to plaque, the amplitude of the peak Ca^2+^ response was significantly greater in thoracic than arch regions (at 0.3, 3 & 10 μmol/L CCh) but the pEC_50_ was unaltered (Fig. 3E). Away from areas of plaque, this difference in amplitude was lost (Fig. 3D).

### Effect of age on CCh response: 10 weeks versus 24 weeks

**Figure 4.**
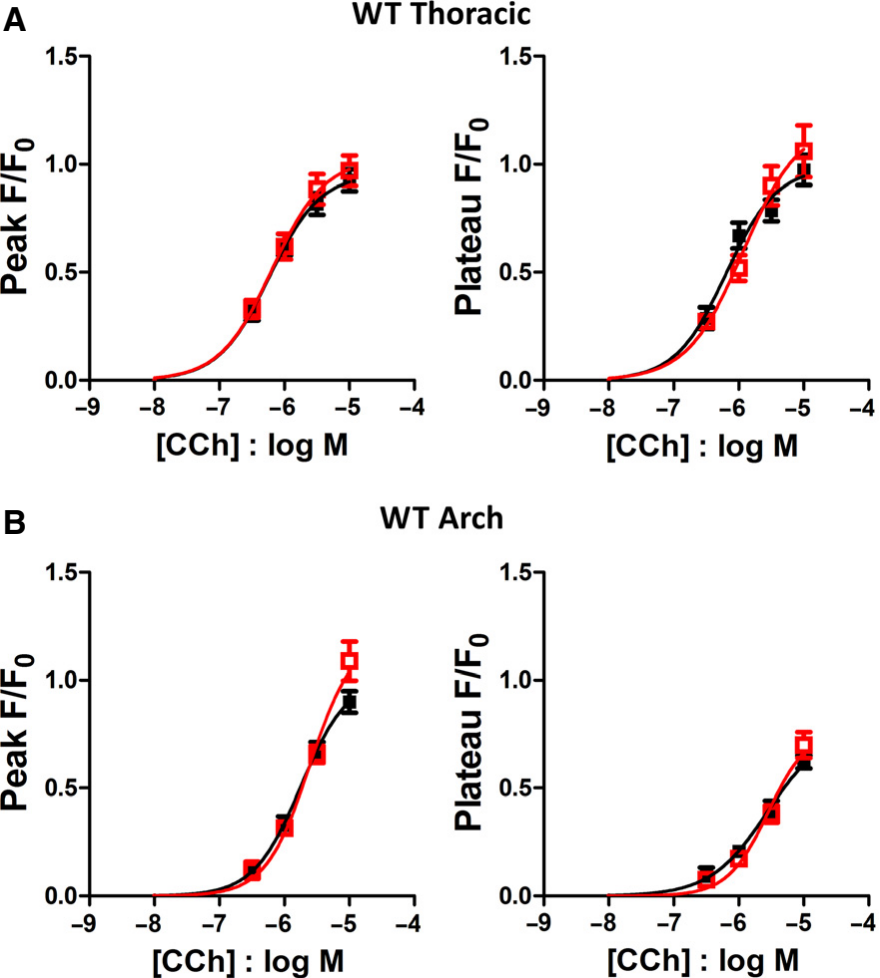
Effect of increasing age on carbachol responses in WT thoracic and aortic arch endothelial cells. Carbachol concentration‐response curves in endothelial cells from 10 week (■) and 24 week old WT mice (), from thoracic aorta (A) and aortic arch (B) sections.

**Figure 5.**
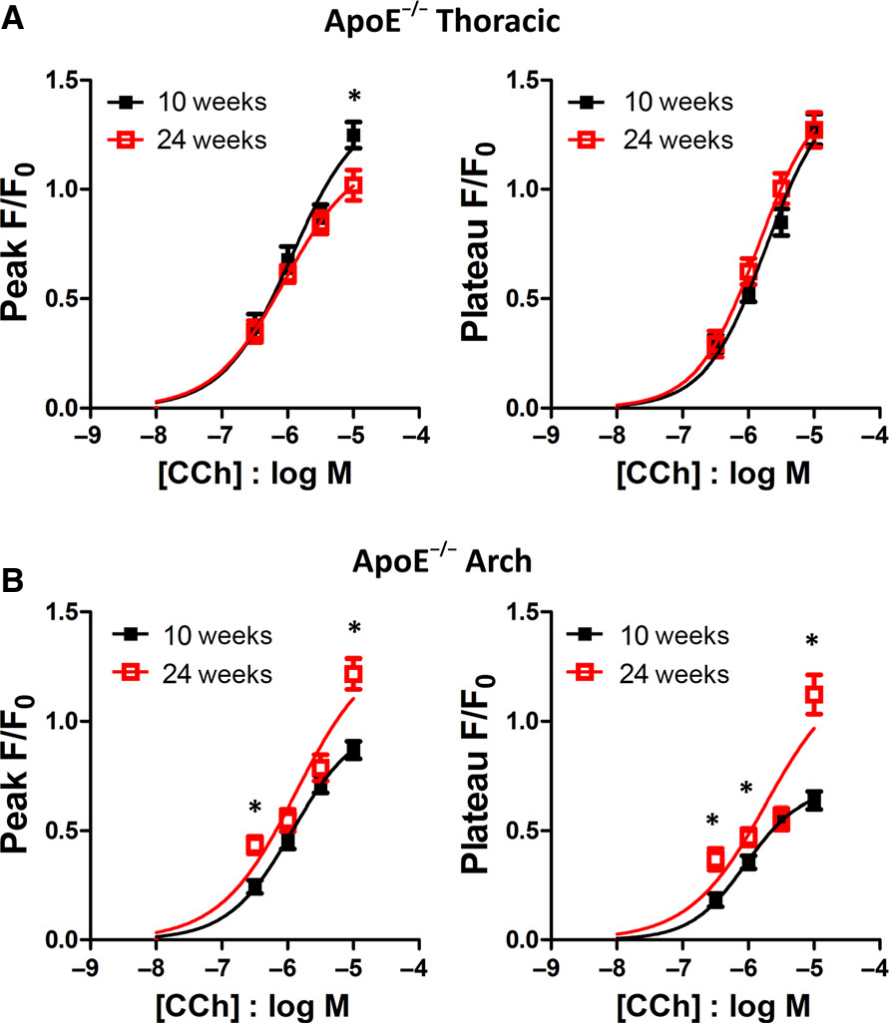
Effect of increasing age on carbachol responses in ApoE^−/−^ thoracic and aortic arch endothelial cells. Carbachol concentration‐response curves in endothelial cells from 10 week (■) and 24 week () old ApoE^−/−^ mice, from thoracic aorta (A) and aortic arch (B) sections. Measurements were from plaque‐free regions in the older mice. *P < 0.05

In order to examine the effects of aging on the CCh concentration‐response curve, all measurements were made from endothelial cells in plaque‐free areas. In WT mice, increasing age did not alter the amplitude or potency of the CCh concentration‐response curves in either thoracic aorta or aortic arch sections (Fig. 4). In thoracic aorta from ApoE^−/−^ mice, minimal or no alterations of the Ca^2+^ peak and plateau responses were observed in 24 week old mice (Fig. 5A), similar to the WT mice. In aortic arch, significantly increased peak and plateau responses were observed at 24 weeks of age (Fig. 5B), presumably the result of hypercholesterolemia‐mediated alterations in Ca^2+^ signalling.

### Effect of plaque development on CCh response

In 24 week old ApoE^−/−^ mice plaques are established and we have measured Ca^2+^ responses in endothelial cells immediately adjacent to plaque and compared them to responses in plaque‐free regions. In both thoracic aorta and aortic arch of old ApoE^−/−^ mice, the presence of plaque failed to alter the amplitude or potency of the CCh response compared to regions without plaque (not shown).

### Effect of hypercholesterolemia

We have compared the CCh concentration‐response curve in WT and ApoE^−/−^ mice in order to investigate the effects of hypercholesterolemia.

#### 10 week old mice

**Figure 6.**
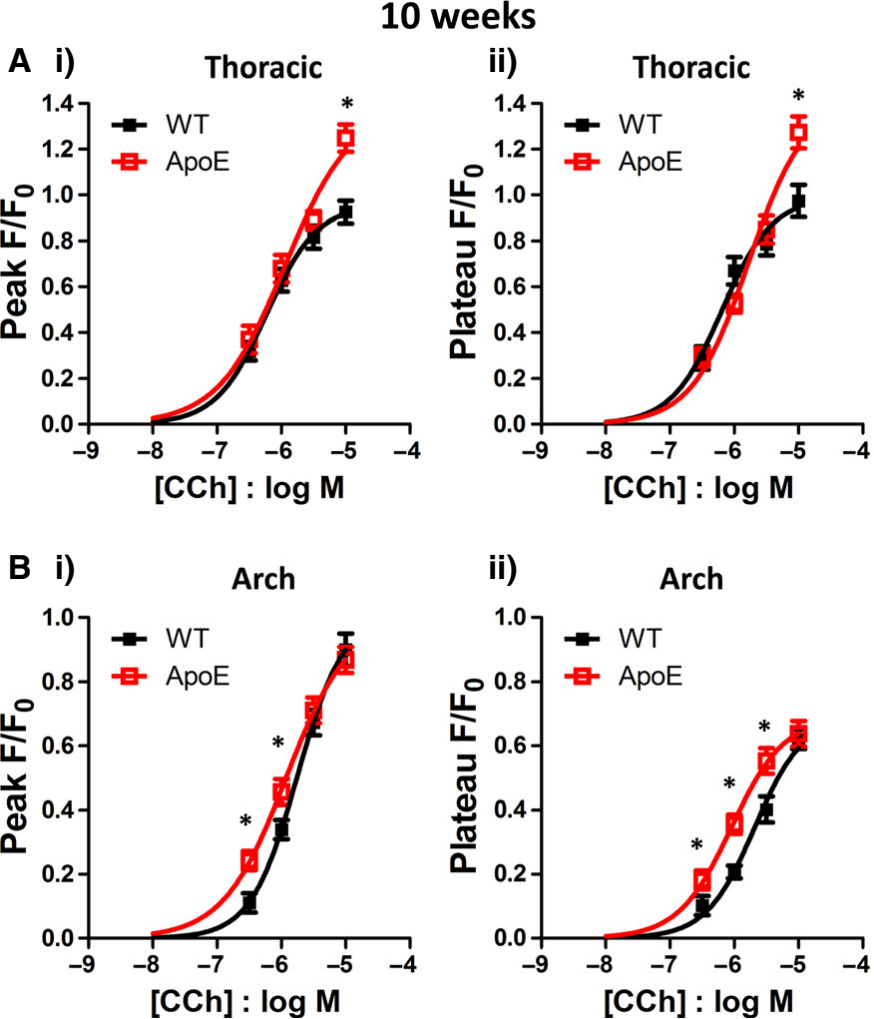
Effect of carbachol on 10 week old thoracic and aortic arch endothelial cells. Carbachol concentration‐response curves in endothelial cells from thoracic (A) and aortic arch (B) sections of 10 week old WT (■) and ApoE^−/−^ () mice. (i) measurement of peak Ca^2+^ response and (ii) measurement of Ca^2+^ plateau response. *P < 0.05.

In aortae from young WT and ApoE^−/−^ mice plaques were absent and therefore all measurements were made from cells in plaque‐free areas. In thoracic aorta, there was no difference in the amplitude of the Ca^2+^ peak and plateau responses in WT and ApoE^−/−^ cells, except at the highest CCh concentration of 10 μmol/L, where the ApoE^−/−^ response was increased compared to WT (*N* = 5–7 mice, Fig. 6Ai and ii). In the aortic arch, the peak Ca^2+^ response was significantly greater in ApoE^−/−^ endothelial cells at 0.3 and 1 μmol/L CCh (*N* = 4–5 mice, Fig. 6Bi) and the size of the plateau was significantly greater at 0.3, 1 and 3 μmol/L CCh (Fig. 6Bii).

#### 24 week old mice

**Figure 7.**
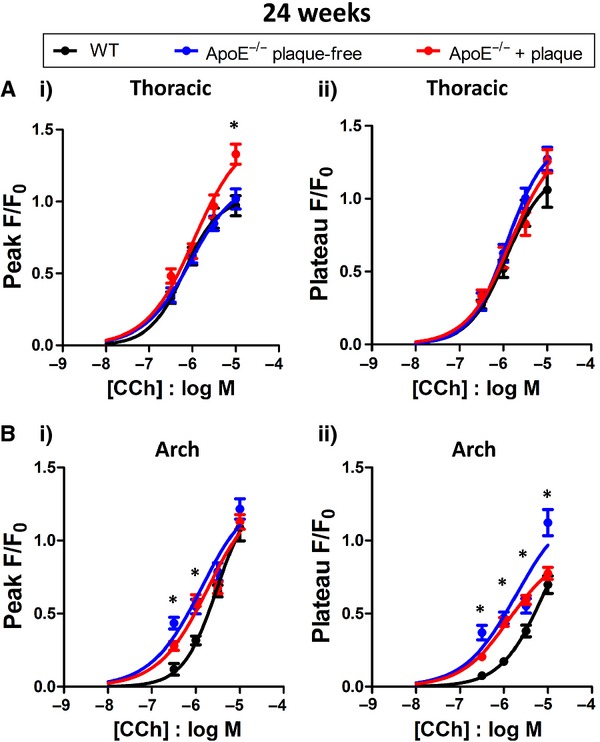
Effect of carbachol on 24 week thoracic and aortic arch endothelial cells. Carbachol concentration‐response curves in endothelial cells from thoracic (A) and aortic arch (B) sections of 24 week old WT (●) and ApoE^−/−^ mice. (i) measurement of peak Ca^2+^ response and (ii) measurement of Ca^2+^ plateau response. In ApoE^−/−^ endothelial cells, measurements were taken either immediately adjacent to plaque () or at a distance from regions of plaque (). *P < 0.05

**Figure 8.**
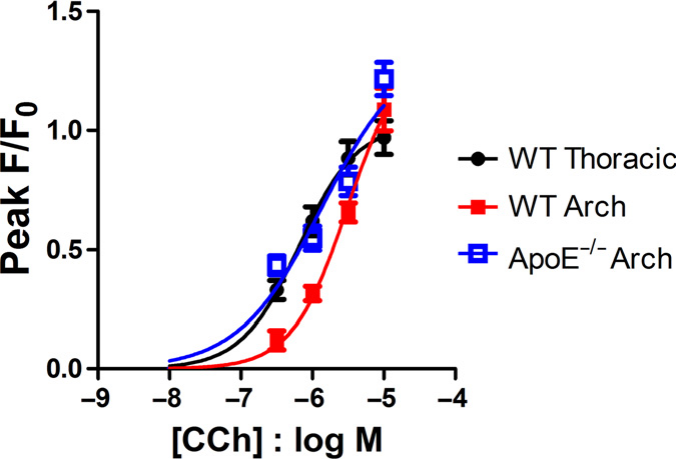
Comparison of carbachol response in 24 week thoracic and aortic arch endothelial cells. Carbachol concentration‐response curves in endothelial cells from WT thoracic (●), and WT () and ApoE^−/−^ () aortic arch sections of 24 week old mice.

Plaques were established in ApoE^−/−^ mice of this age, so measurements were made in endothelial cells both adjacent to and away from areas of plaque. In thoracic aorta from 24 week ApoE^−/−^ mice, the amplitude of the plateau phase of the Ca^2+^ response to CCh was not significantly different to WT either adjacent to or away from regions of plaque (*N* = 3–4 mice, Fig. 7Aii). The amplitude of the peak Ca^2+^ response was not significantly different in ApoE^−/−^ endothelial cells away from plaques, nor adjacent to plaque, apart from a significant increase at 10 μmol/L CCh (*N* = 3–4 mice, Fig. 7Ai), similar to the response seen at 10 weeks of age. In the aortic arch, where the plaque load is greater, the amplitude of the Ca^2+^ peak response was significantly greater in ApoE^−/−^ endothelial cells in response to 0.3 and 1 μmol/L CCh, whether measured directly adjacent to or further away from plaques (Fig. 7Bi) and the Ca^2+^ plateau was significantly greater in ApoE^−/−^ endothelial cells in response to 0.3, 1 and 3 μmol/L CCh (*N* = 3–4 mice, Fig. 7Bii). The increased Ca^2+^ signals in the endothelial cells in ApoE^−/−^ arch resulted in these Ca^2+^ signals now being similar (in terms of amplitude and potency) to those found in WT thoracic cells (see Fig. 8). In the thoracic aorta of 30 week old mice, peak and plateau Ca^2+^ responses were not increased compared to WT in plaque‐free locations, but significantly increased adjacent to plaque (*N* = 3–6 mice, unpublished data).

### Calcium oscillations

**Figure 9.**
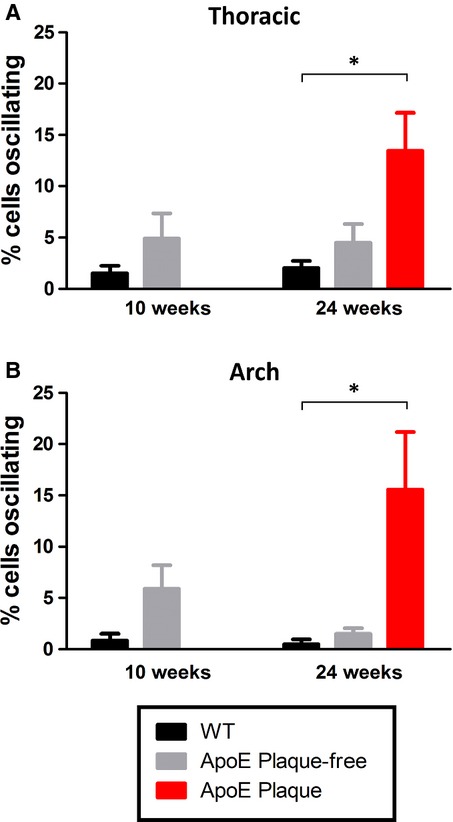
Effect of age and proximity to plaque on the oscillatory Ca^2+^ response. Percentage of WT and ApoE^−/−^ endothelial cells responding to 10 μmol/L CCh with an oscillatory Ca^2+^ response in (A) thoracic and (B) aortic arch, at 10 and 24 weeks of age. In older mice, measurements were taken either immediately adjacent to plaque or from plaque‐free regions. *P < 0.05.

As previously described in Prendergast et al. (2014), a small number of aortic endothelial cells respond to CCh with an oscillatory Ca^2+^ response. In this study, we have examined whether the percentage of cells producing an oscillatory response varies depending on age and location (aortic arch versus thoracic aorta, plaque‐free vs. plaque‐laden). In WT mice of any age, ~1–2% of cells give an oscillatory Ca^2+^ response (see Table 1) and there was no significant difference between thoracic and aortic arch. In young ApoE^−/−^, the number of cells responding with Ca^2+^ oscillations was higher (5–6%, Table 1 and Fig. 9) but did not reach significance and again there was no difference between the thoracic and arch regions. In plaque‐free regions of 24 week old ApoE^−/−^ mice, the percentage of cells producing an oscillatory response was not significantly different from WT (Table 1). Significant increases in the percentage of cells producing an oscillatory response were observed however in 24 week ApoE^−/−^ cells immediately adjacent to plaque (Thoracic 13.4 ±3.7% and Arch 15.6 ± 5.5% (Fig. 9).

## Discussion

Using confocal microscopy, we have examined aortic endothelial cell Ca^2+^ signalling in the apolipoprotein E knockout mouse and determined that age, the location of cells within the vessel and the absence or presence of atherosclerotic plaques determines the size and type of calcium signal observed. In addition, as few data are available on the Ca^2+^ signalling events in endothelial cells of WT mice, we have also detailed these where appropriate.

### Regional dependency of calcium signalling in WT arch and thoracic aortae

Previous data from species including mice had indicated that there was a regional dependency of endothelium‐dependent relaxation along the aorta. These functional studies lead to the conclusion that responses to acetylcholine in thoracic versus abdominal aorta or proximal versus distal sections of thoracic aorta, increase moving down the aorta from proximal to distal sections (Gregg et al. 1995; Honda et al. 1997; Darblade et al. 2002; Horvath et al. 2005; Oloyo et al. 2012). These functional differences have been attributed to differences in the bioavailability of NO, as COX or EDHF inhibition had no effect, whereas the relaxation was abolished if inhibitors of NOS were used. None of these studies however had measured Ca^2+^ signals in the different sections to determine if this underlay the different NOS responses. We found that endothelial cell Ca^2+^ signals in response to CCh were larger in amplitude and more potent in thoracic aorta of both young and old WT mice compared to WT aortic arch. This suggests that even in the absence of dyslipidaemia there are regional differences in Ca^2+^ signals, which in turn are consistent with the well documented greater relaxation of distal thoracic aorta to muscarinic agonists. As discussed next, when we assess data obtained in ApoE^−/−^ mice, differences in the pattern of blood flow through the aorta, may ultimately account for these regional differences.

### Calcium signalling along the aorta differs in ApoE^−/−^ mice

We observed that under conditions of hypercholesterolemia, as with WT mice, the amplitude of the Ca^2+^ signals was still larger in thoracic aorta than aortic arch, but the difference in potency was lost. The aortic arch is a common site of early atherosclerotic plaque development due to its turbulent blood flow (Prado et al. 2008; Zhou et al. 2010), whereas atherosclerosis‐resistant regions are characterised by laminar blood flow but the same dyslipidaemia (Ku et al. 1985; Berceli et al. 1990; Moore et al. 1994; Deng et al. 1995). Together with the lack of turbulent blood flow in the thoracic aorta, a larger, more potent response to CCh may allow the thoracic aorta to maintain better NO production and stave off endothelial dysfunction for longer than the aortic arch and therefore remain relatively resistant to plaque development. A similar conclusion was reached by Chen et al. (2007), who observed that Ca^2+^ signals to ACh were larger in the plaque‐resistant carotid artery of hypercholesterolemic rabbits, than the plaque‐prone aorta.

### The effect of age on calcium signalling

There are differing reports of the effect of age on the functional vascular response to muscarinic‐mediated relaxation in mice, with some studies showing impaired relaxation, as a result of increasing amounts of superoxide radicals (Blackwell et al. 2004; Takenouchi et al. 2009) and others observing no differences (in ApoE^−/−^ mice; Wang et al. 2000). Similarly mixed results are observed in other species (Chinellato et al. 1991; Ishihata et al. 1991; Crespo et al. 1996; Hashimoto et al. 1998; Kano et al. 2000). Endothelial and smooth muscle Ca^2+^ signalling changes can occur with aging (Matz et al. 2003; Goyal et al. 2009; Perrier et al. 2009; Seals et al. 2011). In our studies, CCh‐mediated Ca^2+^ signalling was unaltered with age in WT mice. The regional differences between thoracic and arch sections also remained evident at 24 weeks. In ApoE^−/−^ mice, Ca^2+^ signals remained essentially unaltered with increasing age in the plaque‐resistant thoracic aorta, but were significantly increased in the plaque‐prone aortic arch, presumably as a direct result of the elevated cholesterol levels present in these knockout mice.

Caveolae are cholesterol‐rich microdomains in the plasma membrane that are involved in the compartmentalization of many signalling molecules. Caveolin‐1 (cav‐1) is abundantly expressed in the vasculature, binds cholesterol and is necessary for caveolae formation. An important role for cav‐1 in atherosclerosis has been demonstrated using a cav‐1/ApoE^−/−^ double knockout mouse (Frank et al. 2004), where loss of caveolae resulted in a significant decrease in aortic plaque load. We and others have shown that cholesterol and caveolae are important in the regulation of Ca^2+^ signalling pathways (Darby et al. 2000; Bergdahl et al. 2003; Pouvreau et al. 2004; Cheng and Jaggar 2006; Kamishima et al. 2007; Shmygol et al. 2007; Galan et al. 2010; Prendergast et al. 2010; Pani and Singh 2009 for review). Alterations in cholesterol levels can therefore disrupt normal caveolae‐mediated Ca^2+^ signalling and we surmise that the hypercholesterolemic state present in these ApoE^−/−^ mice is also leading to Ca^2+^ signalling alterations.

### Calcium signalling changes are greater in aortic arch than thoracic aorta

In 10 week old ApoE^−/−^ mice, we observed that hypercholesterolemia differentially affects Ca^2+^ signalling in endothelial cells from aortic arch and thoracic aorta. In thoracic aorta, the Ca^2+^ response was unaltered compared to WT, whereas in aortic arch sections, a widespread, significant increase in the Ca^2+^ response was apparent. This is despite the fact that these 10 week old ApoE^−/−^ mice are not yet exhibiting plaques in the aorta. We have previously shown that this increased response is reversed by treatment with methyl‐β‐cyclodextrin and therefore directly related to the elevated levels of cholesterol present in these knockout mice (Prendergast et al. 2014). Thus this difference between ApoE^−/−^ and WT aortic arch endothelial cells cannot be attributed to differences in flow but rather to the dyslipidaemia.

Once plaques are established, it is clear that greater signalling changes are also observed in aortic arch than in thoracic aorta. Thoracic aorta remains more resistant to the effects of hypercholesterolemia, with few signalling changes seen either in plaque‐free regions or adjacent to plaques. In arch endothelial cells, the susceptibility to hypercholesterolemia and atherosclerosis is seen in a more extensive response to CCh. Similar increases in the size of Ca^2+^ response were seen in 24 week arch sections irrespective of whether responses were measured in cells adjacent to plaques or in plaque‐free regions. The increased endothelial cell Ca^2+^ signals in 24 week old ApoE^−/−^ aortic arch were such that these Ca^2+^ signals were now as potent and the amplitude as large as those found in WT thoracic endothelial cells (Fig. 8). It seems that in these ApoE^−/−^ cells, an effort is underway to compensate for a developing endothelial dysfunction. However, we know from our previously published functional studies (Prendergast et al. 2014), that despite these alterations in Ca^2+^ signalling, the relaxatory response to CCh is compromised even before plaque development, something which was not found to be related to a greater production of superoxide radicals.

The CCh concentration‐response curve data suggest that the plateau phase of the Ca^2+^ response is more susceptible to change than the initial Ca^2+^ peak, which would imply that changes to Ca^2+^ entry rather than Ca^2+^ release are most affected by dyslipidemia. We know SOCE to be decreased in these aortic endothelial cells (Prendergast et al. 2014), so we speculate that the increased Ca^2+^ response to CCh may therefore be the result of a decreased Ca^2+^ efflux (shown to be altered in ApoE^−/−^ vascular smooth muscle by Ewart et al. (2014)) or a decreased ER uptake (Adachi et al. 2002; Guns et al. 2008). Further studies will be required to confirm these suppositions.

### Plaque associated endothelial cell calcium signalling dysfunction

The presence of plaque made minimal difference to the CCh concentration‐response curves in 24 week old ApoE^−/−^ mice. Ca^2+^ responses were largely unaltered in thoracic aorta and significantly enhanced in aortic arch irrespective of the presence of plaque. This is in opposition to the findings of Crauwels et al. (2003) and Guns et al. (2008), who observed an endothelial dysfunction only immediately adjacent to plaques, not in plaque‐free regions. However, preliminary unpublished observations from our laboratory suggest that at 30 weeks, Ca^2+^ responses to CCh in the thoracic aorta are enhanced adjacent to plaque but not in plaque‐free regions, suggesting perhaps that a more extensive plaque load may eventually begin to alter the Ca^2+^ response in this more plaque‐resistant area.

### Increase in calcium oscillations

In addition to alterations in the amplitude of the response to CCh in ApoE^−/−^ mice, we also observed that hypercholesterolemia led to Ca^2+^ signals becoming more oscillatory in nature, particularly in aged mice and immediately adjacent to areas of plaque. The Ca^2+^ response that results from stimulation with CCh is the result of a complex series of cellular processes: Ca^2+^ release from the internal store, Ca^2+^ re‐uptake by SERCA pumps, Ca^2+^ extrusion via PCMA and NCX and Ca^2+^ entry via store‐operated Ca^2+^ channels, receptor‐operated channels or non‐selective cation channels (Wang et al. 1995; Wang and van Breemen 1997; Nilius and Droogmans 2001; Boittin et al. 2008). The literature indicates that many of these processes are sensitive to an alteration in cholesterol levels. Our own data showed SOCE to be reduced in ApoE^−/−^ endothelial cells (Prendergast et al. 2014). Other studies in ApoE^−/−^ vascular smooth muscle cells showed increased IP_3_‐mediated release of Ca^2+^ from the store (Van Assche et al. 2007), reduced SERCA expression and increased PMCA expression (Ewart et al. 2014) and increased SOCE (Van Assche et al. 2007). Many other models of hypercholesterolemia or models where cholesterol levels are modulated using tools such as MCD also demonstrate these processes to be susceptible to changes in cholesterol levels (Adachi et al. 2001; Bergdahl et al. 2003; Murata et al. 2007; Galan et al. 2010; Shiraishi et al. 2013). In old ApoE^−/−^ mice, adjacent to established plaques, cholesterol levels must locally be particularly elevated, leading to the significant alterations in Ca^2+^ handling that manifests itself as an increase in oscillatory Ca^2+^ responses to CCh. Although not directly investigated in this study, we speculate that it is changes induced by altered cholesterol levels that are affecting membrane microdomains and caveolae specifically. As we (Prendergast et al. 2010) and others (e.g. Dreja et al. 2002 and Cristofaro et al. 2007) have shown, disruption of caveolae produces profound but selective effects on cell signalling, with muscarinic receptors being clearly affected (Lai et al. 2004; Gosens et al. 2007; Schlenz et al. 2010; Ekman et al. 2012; Bhattacharya et al. 2013). We have previously speculated that the switch from a sustained Ca^2+^ response to a more oscillatory response in endothelial cells may lead to less effective stimulation of NO formation and thus explain the dichotomy in ApoE^−/−^ mice of elevated Ca^2+^ responses but reduced CCh‐mediated relaxation as described in Prendergast et al. (2014). Further studies are required to verify this hypothesis.

### Relevance to atherosclerosis

We have demonstrated clear alterations in Ca^2+^ signalling both before and subsequent to development of plaques in the plaque‐prone aortic arch but much fewer signalling differences in the plaque‐resistant thoracic aorta. Endothelial cells exposed to disturbed blood flow show upregulated mRNA levels of pro‐atherogenic genes (proinflammatory, proapoptotic, procoagulant genes; Brooks et al. 2002; Dai et al. 2004), whereas endothelial cells in regions with laminar flow express atheroprotective genes (SOD1, NOS; Wasserman and Topper 2004). The involvement of calcium signalling pathways in the development of atherosclerosis has been previously recognised. Yuan et al. (2009) used a microarray expression profiling approach, examining gene expression in aortic walls of ApoE^−/−^ mice and identified the calcium signalling pathway as being implicated in the control of atherosclerosis susceptibility. Similarly, in Van Assche et al. (2010), transcription profiles of modifiers of SERCA function were found to be differentially regulated in plaque‐prone aortic smooth muscle compared to plaque‐resistant regions prior to plaque development. The combination of atherosusceptible gene changes and hypercholesterolemia leads to the significant Ca^2+^ signalling changes that we see in ApoE^−/−^ aortic arch.

### Summary

We have observed that (1) there is a regional variation in Ca^2+^ signalling in WT mice, that is modified in the hypercholesterolemic animals, (2) increasing age does not alter the Ca^2+^ response in WT mice, so the increased response to CCh in older ApoE^−/−^ mice is attributable to hypercholesterolemia, (3) Ca^2+^ signalling changes are greater in endothelial cells from plaque‐prone aortic arch than plaque‐resistant thoracic aorta, (4) CCh concentration‐response curves were unaffected by plaque formation, but (5) Ca^2+^ signals become more oscillatory with age and in particular with plaque formation in ApoE^−/−^ mice.

In conclusion, Ca^2+^ signalling changes occur very early on in the development of atherosclerosis and are more pronounced in plaque‐prone areas than plaque‐resistant areas. Calcium signals become more oscillatory in nature as plaques develop, which may have implications for how well those calcium signals can stimulate generation of nitric oxide. By determining which specific aspects of Ca^2+^ signalling are first susceptible to atherosclerotic progression, we might gain insight into which points in the pathway may be therapeutically worthwhile targeting.

## Conflicts of Interest

No conflicts of interest, financial or otherwise, are declared by the authors.
